# Interaction of healthcare worker hands and portable medical equipment: a sequence analysis to show potential transmission opportunities

**DOI:** 10.1186/s12879-017-2895-6

**Published:** 2017-12-28

**Authors:** Chetan Jinadatha, Frank C. Villamaria, John D. Coppin, Charles R. Dale, Marjory D. Williams, Ryan Whitworth, Mark Stibich

**Affiliations:** 1 Central Texas Veterans Health Care System, 1901 Veterans Memorial Drive, Temple, TX 76504 USA; 2 Department of Medicine, College of Medicine, Texas A&M Health Science Center, 8447 Riverside PKWY, Bryan, TX 77807 USA; 3 Department of Research, Central Texas Veterans Health Care System, 1901 Veterans Memorial Drive, Temple, TX 76504 USA; 4 Xenex Healthcare Services, 121 Interpark, Suite 104, San Antonio, TX 78216 USA

**Keywords:** Portable medical equipment, Sequence analysis, Healthcare-associated infections, Transmission

## Abstract

**Background:**

While research has demonstrated the importance of a clean health care environment, there is a lack of research on the role portable medical equipment (PME) play in the transmission cycle of healthcare-acquired infections (HAIs). This study investigated the patterns and sequence of contact events among health care workers, patients, surfaces, and medical equipment in a hospital environment.

**Methods:**

Research staff observed patient care events over six different 24 h periods on six different hospital units. Each encounter was recorded as a sequence of events and analyzed using sequence analysis and visually represented by network plots. In addition, a point prevalence microbial sample was taken from the computer on wheels (COW).

**Results:**

The most touched items during patient care was the individual patient (850), bedrail (375), bed-surface (302), and bed side Table (223). Three of the top ten most common subsequences included touching PME and the patient: computer on wheels ➔ patient (62 of 274 total sequences, 22.6%, contained this sequence), patient ➔ COW (20.4%), and patient ➔ IV pump (16.1%). The network plots revealed large interconnectedness among objects in the room, the patient, PME, and the healthcare worker.

**Conclusions:**

Our results demonstrated that PME such as COW and IV pump were two of the most highly-touched items during patient care. Even with proper hand sanitization and personal protective equipment, this sequence analysis reveals the potential for contamination from the patient and environment, to a vector such as portable medical equipment, and ultimately to another patient in the hospital.

**Electronic supplementary material:**

The online version of this article (10.1186/s12879-017-2895-6) contains supplementary material, which is available to authorized users.

## Background

High-touch surfaces in the hospital environment, such as bed rails, tray tables, and supply carts, are considered important in the epidemiology of transmission of healthcare-associated infections (HAIs) [1–4]. It has been shown that patients that occupy hospital rooms where the previous occupant was positive for a pathogenic organism have 40% increased odds of transmission than those that occupy rooms where the previous occupant was negative for the pathogenic organism [5]. Since there is no direct contact between the two patients, the risk can be attributed to an environment that is significantly contaminated. Following terminal disinfection, over 50% of surfaces are missed completely during manual cleaning, and 40% of high-touch surfaces sampled have been determined to be inadequately disinfected [6, 7]. If not removed adequately, pathogens can remain viable on fomites for months, serving as a source of transmission on several susceptible patients [8].

An additional source of transmission for which there is limited available research is portable medical equipment (PME). PME includes devices such as computers on wheels (COW), vitals machines, intravenous (IV) infusion pumps, and other appliances intended to be used on multiple patients consecutively [9]. One study examining potential spread of DNA markers between patient room surfaces determined that contamination spread to 100% of PME [10]. A systematic review conducted in 2004 found that over 85% of medical equipment was considered contaminated, with an average of 82.1 colony forming units (CFU) per sample taken from the equipment surfaces [11].

Most PME falls under the noncritical patient care device category of the Spaulding Classification Scheme for infection risk [12]. The disinfection of equipment, along with room high-touch surfaces, is one of the highest priorities in the current Joint Commission scores standard 02.02.01 (Joint Commission) with high non-compliance issues [13]. The Centers for Disease Control and Prevention (CDC) recommend that noncritical patient care devices be cleaned on a regular basis but the recommendations are based on time since the last cleaning rather than how frequently the PME is touched/used [14]. For example, the CDC recommends that a COW be cleaned once a day or as needed, or an IV pump cleaned following patient discharge or disuse. If contact events or “touches” are a means of spreading contamination across surfaces in the environment, then cleaning recommendations based on number of touches may be more effective than those based on the passage of time. However, while existing data on touch frequency have helped to identify high-touch surfaces in the patient care environment, it is difficult to continuously track the patterns of touches. Further research is necessary to quantify the degree of surface contamination associated with touch activity.

While a contaminated environment is a significant contributor to infection, the specificity and regularity to disinfect high-touch surfaces and equipment is warranted. Understanding the complex patterns of interactions involving high-touch surfaces, PME, patient contact, hand-hygiene of healthcare workers, and disinfection of equipment would allow for greater epidemiological understanding of how to contain spread of disease in hospitals. The purpose of this study was to investigate the patterns and sequence of touch events among health care workers, patients, surfaces, and equipment in the hospital environment, to better inform our understanding of potential infection transmission pathways.

## Methods

This observational study was conducted at a 120-bed Veterans Affairs (VA) hospital in Temple, TX, on six inpatient units (four acute medical/surgical units and two intensive care units [ICU]). The acute care units are designated as medical telemetry, medical oncology, mixed medical, and mixed medical-surgical. The ICUs are designated as medical and surgical. Continuous 24-h observation was performed separately on each unit by two research team members observing for 8-h sessions to have consistency in observation recordings. The observers followed one healthcare worker (HCW) for their entire shift, documenting surfaces touched (hard surfaces plus bedding and any PME). During the observation period if additional HCWs entered the room to provide care for the patients [physicians, trainees, food and nutrition workers, therapists (physical, occupational and respiratory), environmental services workers, and mid-level providers], their contact with surfaces and equipment were also captured. The HCW was aware of the observation and recording and was asked to go about normal business and care processes when interacting with the patient. However, the HCW was not informed of the specific data being collected or the purpose of the study beyond the general explanation of observing interactions between people and the environment. Research personnel were known by all HCW to be part of the research team and were accustomed to having team members around. Portable medical equipment used in our facility can be broadly categorized into 2 varieties: PME shared among patients regularly such as vital signs machine, COW, Glucometer, bladder scanners; and PME that stays with the patient from admission to discharge such as IV pump/pole, tray table, wound vacuum. In our medical and surgical intensive care units, we have wall mounted computers and vitals recording machines but COWs are available for use.

### Summary of observation recordings

A patient encounter was defined as follows: initiated when the HCW entered a patient room and completed when the HCW exited the room after the care episode. An observation was defined as a single touch within an encounter, while a sequence was defined as a string of observations during an encounter. For example, patient to COW then to bedrail & IV pump. Observation data recording was limited to HCW such as nurses, physicians, allied health personnel, housekeeping, and food services; patients and visitors were excluded. Observations were not conducted in bathrooms to protect patient privacy. The observations were recorded sequentially throughout the day on a template designed to document the sequence of touches throughout each patient interaction (see Additional file 1). A touch was defined as any contact event between HCW and patient, surface, or equipment recorded in real time along with the sequence of the touches. If more than one HCW was in the room at the same time, touches were recorded as they occurred and notation made as to HCW 1, 2, or 3 respectively but were combined as one HCW for analysis. Observations were recorded for both contact-isolation and non-isolation rooms.

Data included: (1) surface/medical equipment touched, (2) order of touches, (3) what the equipment was used for in that interaction (e.g., a COW could be used for care documentation on the computer or as a surface work area for IV fluids or medications), (4) whether equipment entered or exited the room – to determine if the equipment is patient dedicated or shared (5) if disinfection of equipment or surfaces took place at any time during this interaction, and (6) if hand hygiene was performed.

A list of frequently touched surfaces (patient, high-touch surface or PME) was created, based on the template data. All surfaces touched by HCWs were documented, including surfaces not explicitly listed on the observation template. Only those surfaces/items that had five or more touches over 24 h were included in the sequence analysis.

### Infection prevention activities

The observed activities included donning and doffing of gloves, hand sanitization (either washing with soap and water or using waterless hand sanitizer), and PME disinfection (wiping with disinfectant wipes). This does not factor in discharge or cleaning at change of shift.

### Bioburden estimates

To identify the bioburden on the most common PME (COW), all COWs in use across the hospital were sampled by the research team for aerobic bacterial colonies (ABC) and methicillin-resistant *Staphylococcus aureus* (MRSA) on a single day. The sampling was performed using contact Rodac plates (Hardy Diagnostics, Santa Maria, CA) on the flat table surface and the handle rail of the COW, as well as the scanner used for the medication administration system. The surface area for ABC was 25cm^2^ and for MRSA was 75 cm^2^. The sampling method, incubation, and identification of the organisms are described elsewhere [15].

### Analysis

The average number of touches per encounter was calculated for each surface type and inpatient unit. Sequences were also separated by whether hand hygiene (glove or hand sanitization) occurred (yes/no), and by room access: upon room entry, during the sequence, or upon room exit. We combined the two because both these events potentially represented reduced transmission risk. The observations of touches were recorded as a sequence of events analyzed with sequence analysis software and visually represented by network plots. The network analysis was completed using the package ‘igraph’ in R version 3.2.3 [16]. The sequences were also analyzed for common sub-sequences that might indicate unique/important patterns in HCW interactions with the patient and the environment. This information is visually quantified by the network plots and provides additional details on the characteristics of individual sequences. Sequence mining and analysis were completed using the package ‘TraMineR’ in R version 3.2.3 [17, 18].

## Results

Out of 144 total hours of observation, there were 274 sequences. These sequences varied in length from 1 to 98 touches (mean = 12 .9, median = 8, IQR = 9). Among all observation sequences, 151 (55.1%) of them involved movement of PME in and out of the room.

### Frequently touched surfaces

The top ten items most commonly touched in the patient room (Table 1) were patient with 850 touches, COW (634), bedrail (375), IV pump (326), bed surface (302), tray table (223), vitals machine (213), wall shelf (110), door (90), and in-room computer (78).

**Table 1:** Touch counts for each of 10 surfaces, per encounter

	# encouters (N)	Patient (%)	Bed-surface (%)	Bedrail (%)	Wall shelf (%)	Tray table (%)	Door (%)	In-room Computer (%)	Vitals Machine (%)	Computer On Wheels (%)	Intravenous Pump (%)
Medical oncology	49	149 (21.0)	38 (5.4)	50 (7.0)	23 (3.2)	50 (7.0)	13 (1.8)	0 (0.0)	61 (8.6)	205 (28.9)	106 (14.9)
Mixed medical	35	168 (28.1)	36 (6.0)	49 (8.2)	11 (1.8)	22 (3.7)	13 (2.2)	0 (0.0)	60 (10.0)	184 (30.8)	47 (7.9)
Mixed medical-surgical	52	157 (30.3)	30 (5.8)	58 (11.2)	16 (3.1)	29 (5.6)	25 (4.8)	0 (0.0)	34 (6.6)	113 (21.8)	37 (7.1)
SICU	34	123 (31.6)	38 (9.8)	60 (15.4)	4 (1.0)	52 (13.4)	0 (0.0)	41 (10.5)	3 (0.8)	10 (2.6)	37 (9.5)
MICU	74	194 (25.1)	140 (18.1)	133 (17.2)	42 (5.4)	42 (5.4)	0 (0.0)	37 (4.8)	14 (1.8)	45 (5.8)	82 (10.6)
Medical telemetry	30	59 (18.4)	20 (6.3)	25 (7.8)	14 (4.4)	28 (8.8)	39 (12.2)	0 (0.0)	41 (12.8)	77 (24.1)	17 (5.3)
Sum Totals	274	850 (25.7)	302 (9.1)	375 (11.3)	110 (3.3)	223 (6.7)	90 (2.7)	78 (2.4)	213 (6.4)	634 (19.2)	326 (9.9)
Average Touches per Encounter		3.1	1.1	1.4	0.4	0.8	0.3	0.7^a^	0.8	2.3	1.2

^a^The In-room Computer was only in the ICU, so the average number of touches per encounter was calculated using the total number of encounters from the ICU only

### Infection prevention activities

Donning or doffing of gloves occurred 1.1 times per sequence on average, indicating gloves were routinely used in only about half the encounters. Similarly, hand sanitization was only performed on an average 1.2 times per encounter although it might be expected to occur at both entry and exit. Disinfection of PME by HCW upon entry or exit from room was observed to occur only 17 times over the 144 h of observation. Results are summarized in Table 2.

**Table 2:** Number of times infection control practices were observed and the average number of times each practice occurred per encounter

	# of encounters (N)	Gloves (%)	Hand Sanitization (%)	PME Disinfection (%)
Medical oncology	49	58	61	0
Mixed medical	35	50	50	9
Mixed medical-surgical	52	46	70	2
SICU	34	40	30	1
MICU	74	75	69	2
Medical telemetry	30	42	47	3
Sum Totals	274	311	327	17
Average per Encounter		1.1	1.2	0.06

Two-hundred and thirty of the 274 sequences (83.9%) included hand sanitization or the use of gloves. The most common points at which hand sanitization/glove use occurred were at entry and exit (79.8% of sequences contained hand sanitization/glove use at entry, exit, or both entry and exit).

### Network plots

**Fig. 1:**
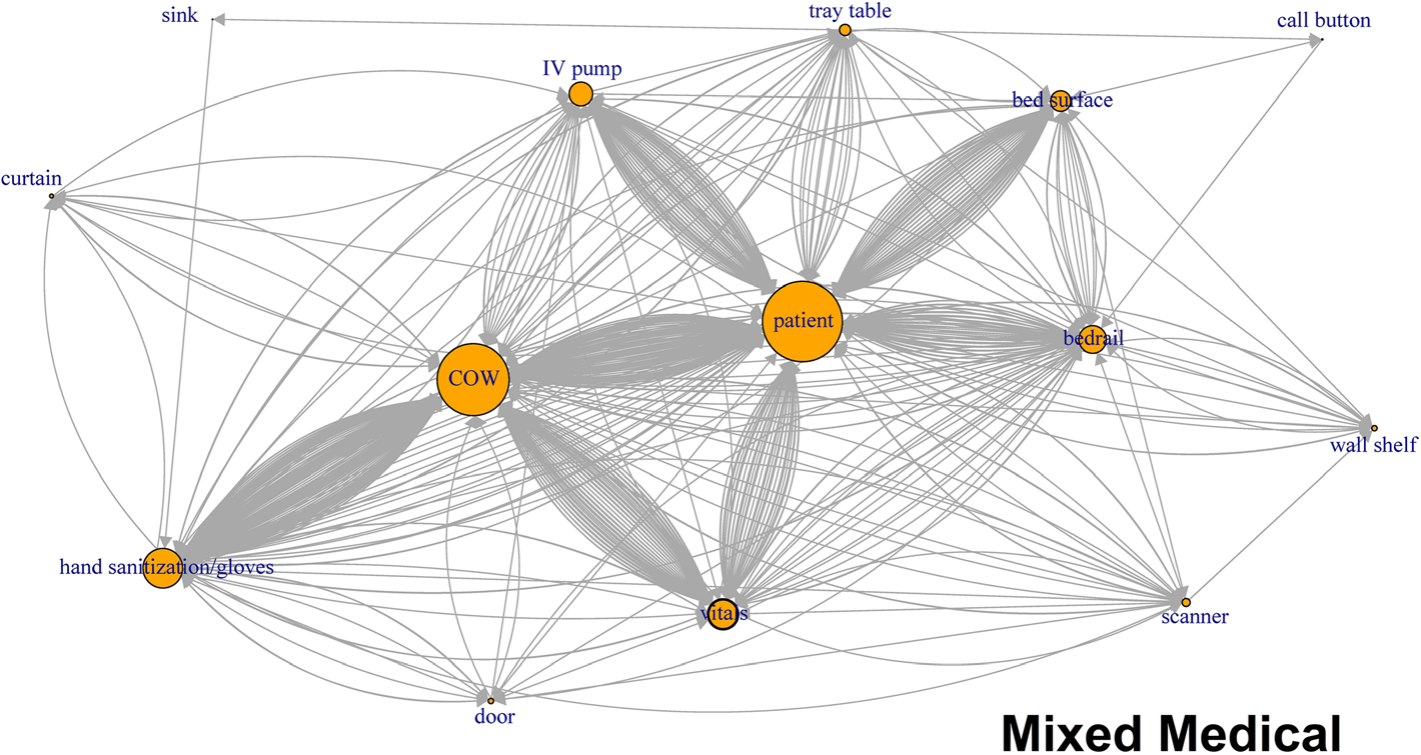
Directed network plots for each inpatient unit aggregating all sequences across all encounters for 24 h of observation

**Fig. 2:**
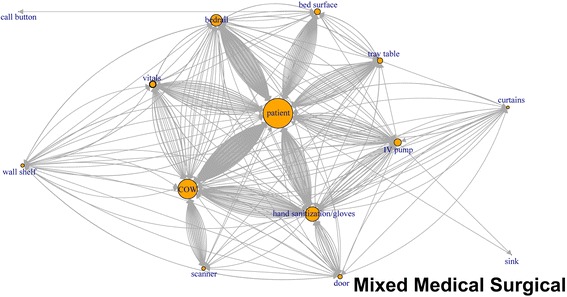
Directed network plots for each inpatient unit aggregating all sequences across all encounters for 24 h of observation

**Fig. 3:**
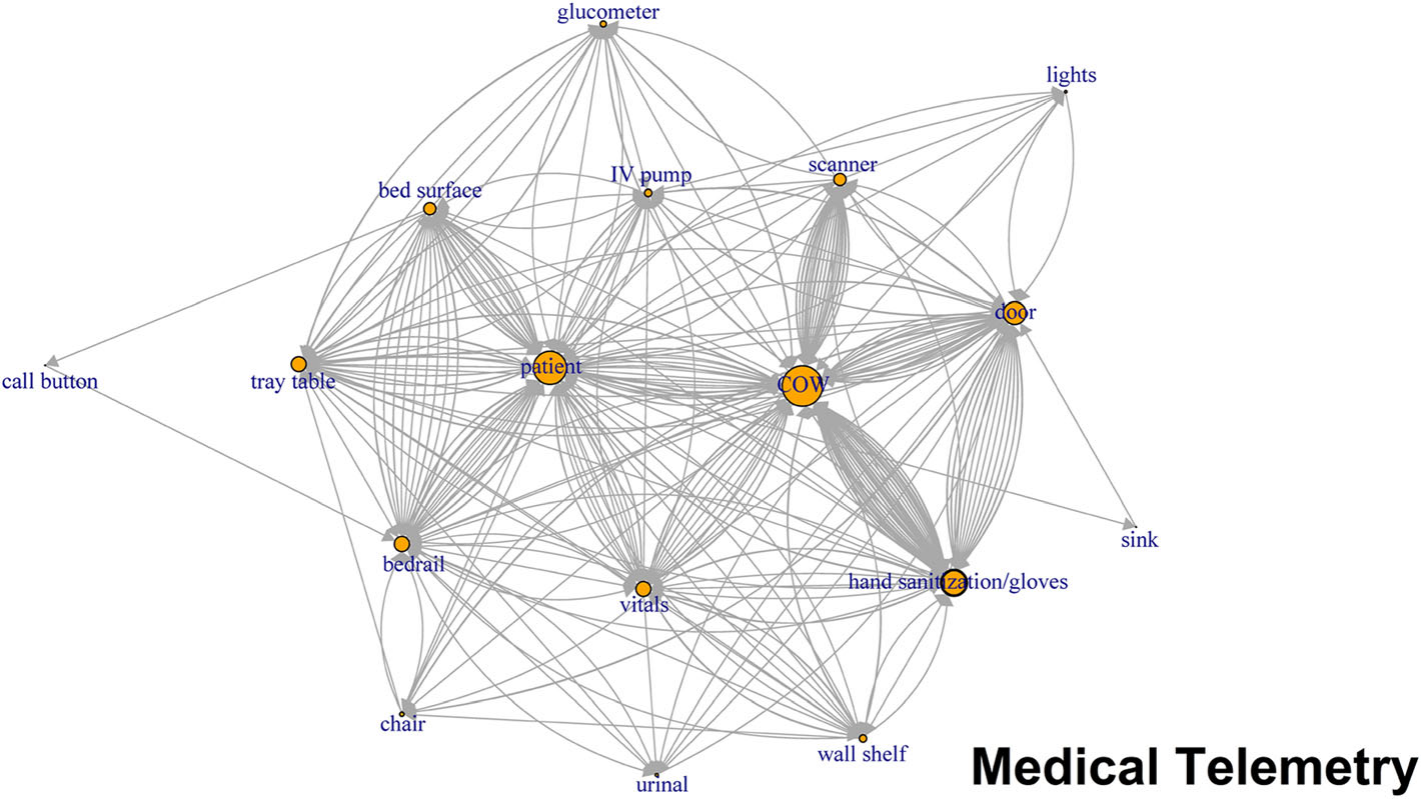
Directed network plots for each inpatient unit aggregating all sequences across all encounters for 24 h of observation

**Fig. 4:**
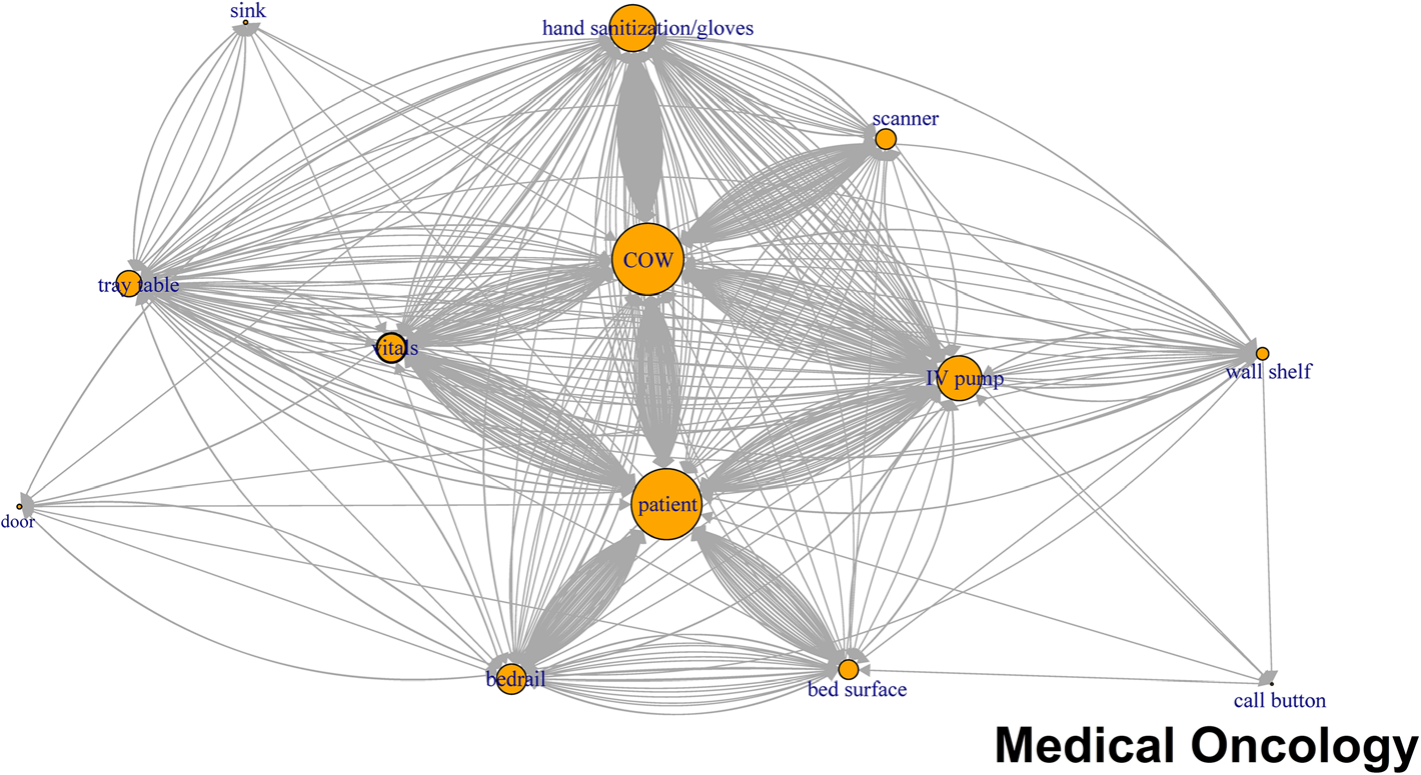
Directed network plots for each inpatient unit aggregating all sequences across all encounters for 24 h of observation

**Fig. 5:**
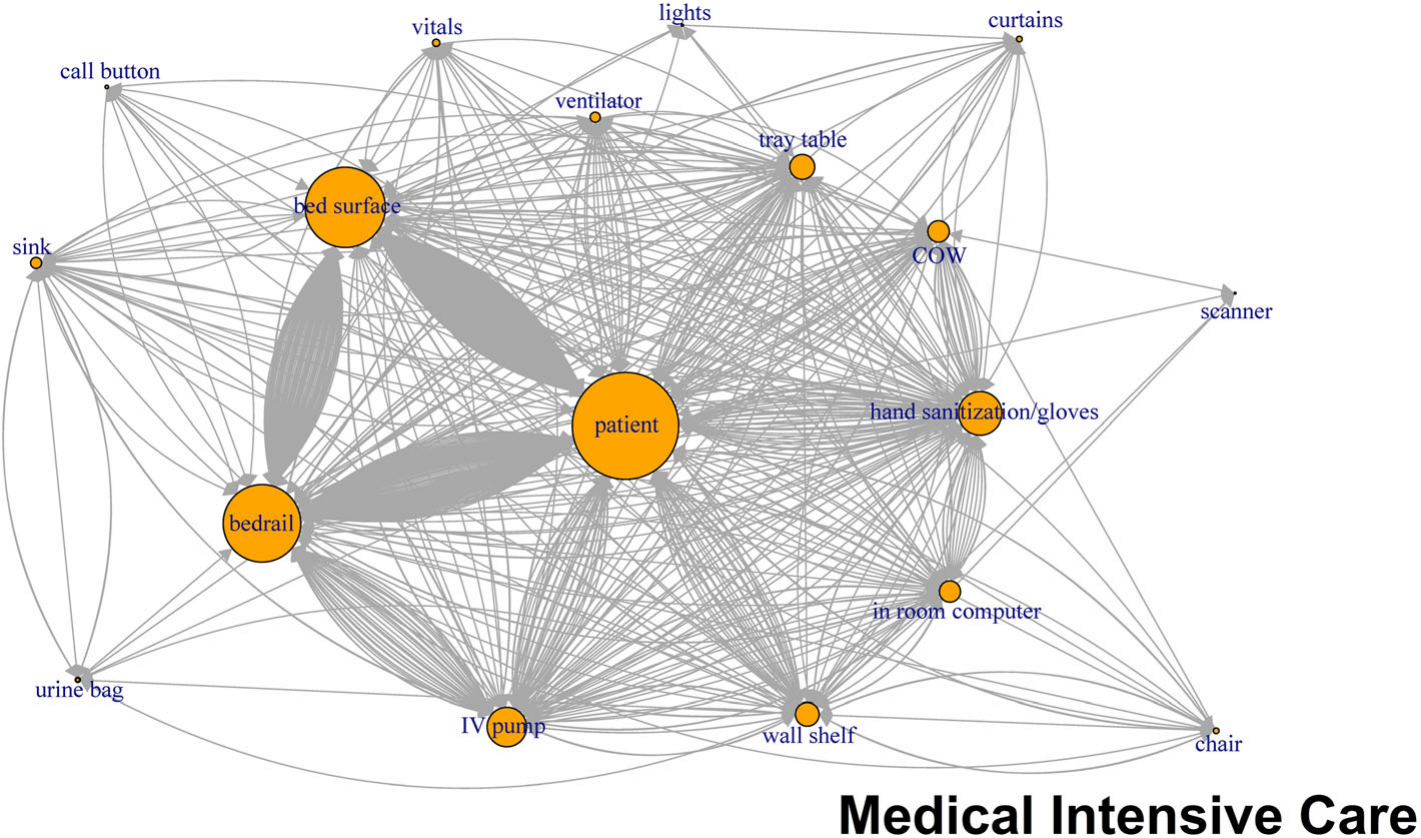
Directed network plots for each inpatient unit aggregating all sequences across all encounters for 24 h of observation

**Fig. 6:**
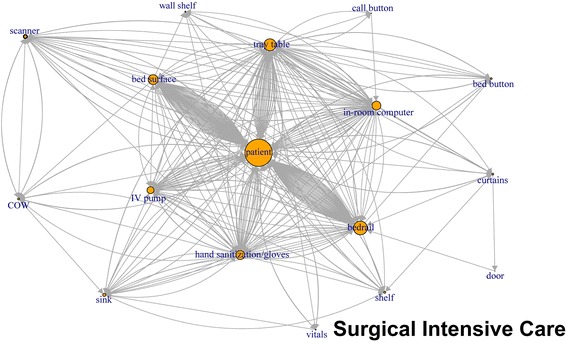
Directed network plots for each inpatient unit aggregating all sequences across all encounters for 24 h of observation

Like application of network plots to depict social networks, our plots of touch sequences delineate objects that play the most central role in a sequence of touches, by describing both the volume of touches and other objects that are immediately connected by the sequence of touches (Figs. 1, 2, 3, 4, 5, 6). The circles in the network plots indicate a central object that was touched (including a patient). The arrows indicate the order in which the objects were touched (i.e., sequence). The density of lines between objects indicates the number of times the touch sequence occurs. The arrow indicates the direction of the touch sequence, e.g., the HCW touched the IV pump and then the COW.

In Fig. 1 (mixed medical), the center of the plot is the patient and COW, the two most-touched surfaces. The figure depicts the touch order with patient being touched before or after the bed rail, bed surface, vitals machine, or IV pump tray table. Similarly, the COW was also touched often before and after the following objects: patient, vitals machine, IV pump hand sanitizer, privacy curtain.

### Sequence analysis

**Fig. 7:**
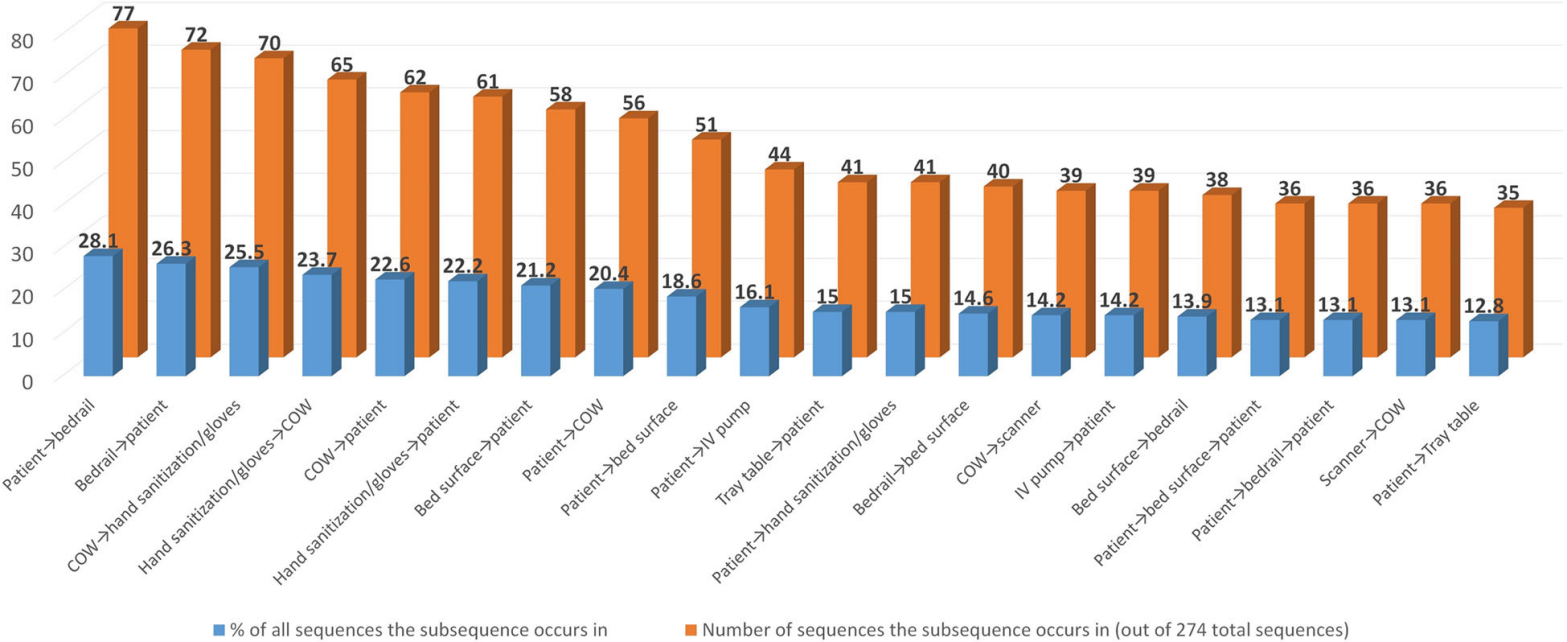
The 20 most common sub-sequences of touches in the patient room or ICU over a 24-h observation period

Sequence analysis was performed to analyze the order in which the objects, patients, and surfaces were touched. The analysis might provide an insight into common sources of transmission and prevention possibilities. Touching the patient and then the bedrail was the most common sub-sequence, occurring at least once in 77 of the 274 sequences (28.1% of all sequences) (Fig. 7). Bedrail ➔ patient was the second most common sub-sequence. The next two most common sub-sequences overall involved PME. Touching the COW and then putting on gloves and/or sanitizing hands (25.5% of all sequences contained this sub-sequence) was third, while putting on gloves and/or sanitizing hands and then touching the COW was the fourth most common sub-sequence. Touching the COW and then the patient was the most common sub-sequence between PME and patient (22.6% of all sequences contained this sub-sequence). Only two sub-sequences longer than two touches were among the 20 most commonly occurring sub-sequences: touching the patient followed by the bed surface then the patient again (in 13.1% of sequences), and touching the patient then the bedrail then the patient again (also 13.1%). Fourteen of the twenty most commonly occurring sub-sequences involved touching the patient, eight involved PME, and 8 involved a surface on the bed. Hand sanitization and/or glove use occurred in 4 of the 20 most commonly occurring sub-sequences.

### Bioburden estimates

The average count for ABC was 31 CFU/25 cm^2^ (Table 3). The hand rail of the COW was more contaminated than the scanner or the table portion of the COW. Medical oncology ward had the lowest ABC counts and the mixed medical-surgical ward had the highest. Five out of 48 COW’s (10.4%) had MRSA. MRSA was found on the table of 1 COW with 2 CFU, on the rail of 3 COW’s with a total of 5 CFU and on the scanner of 1 COW for a total count of 1 CFU.

**Table 3:** Aerobic bacterial colony counts from 3 areas of computer on wheels

Surface Location on a COW	Min.	1st Qu.	Median	Mean	3rd Qu.	Max.
Table	0	10.8	20	29.8	32 .5	177
Handrail	4	11.8	21.5	40.9	48.5	304
Scanner	0	4	8.5	21.5	18	218
Floor	Min.	1st Qu.	Median	Mean	3rd Qu.	Max.
Medical Telemetry	0	10	19.5	25.5	34.5	69
Intensive Care Unit	1	11	17	33.7	32.8	218
Medical Oncology	0	6	11	20.1	19	174
Mixed Medical-surgical	3	12	29.5	51.2	49	304
Mixed Medical	3	6	11	26.2	20.5	239

## Discussion

As might be expected, the patient was the common thread in almost all the interactions and was touched the most frequently in all our observations. PME, particularly COW and IV pump, are highly touched items and were common in sub-sequences that involved the patient, possibly contributing to higher pathogen transmission risk. An overwhelming density of touches can be seen to and from the COW and patient, leading the observer to believe that cross transmission is very plausible between these two surfaces. Despite the availability of in room computers and vitals machine in ICUs we observed HCWs still using COWs without regular disinfection. Previous studies have described the frequently touched surfaces in a hospital environment by healthcare workers [4]. Another study described the common contamination of portable medical equipment by patients [19]. However, to the authors’ knowledge, no previous study has sought to identify the sequence of HCW touches or to map the connections between surfaces touched and portable medical equipment. The surfaces we identified as high-touch are similar to what has been described in Huslage et al. [4]. They are bedrails, bed surface, and IV pump in the ICU (Huslage also identified the supply cart, which our ICU did not have) [4]. Other studies have identified bedrail as the most frequently touched surface [20]. Our results differed slightly from Huslage et al. for the medical and surgical floors, where we found that the three most-touched surfaces were COW, bedrails and IV pump as opposed to bedrails, tray table, and bed surface [4]. Adams et al. showed that increased bioburden is correlated with more frequent touches to near-bed surfaces in the ICU [21]. If the HCW and PME are viewed as possible vehicles for transmission, then the connections between other surfaces, the HCW, and PME become possible routes of pathogen transmission. Surfaces that have high connectivity in the sequence of touches, or are part of common sub-sequences performed by HCWs, may be critical points along the routes of contamination. Identifying and addressing contamination at these points in the route may help to decrease the probability of transmission of pathogens from the environment to the patient and vice versa.

Observed hand hygiene in this study was below expectations despite the possible Hawthorne effect from HCWs knowing that Infection Control research staff were observing them. Poor hand hygiene can increase contamination of clean PME as well as risk of cross-contamination among surfaces. Thus, hand hygiene needs to be considered in the context of equipment cleanliness.

Hospital policy designates that COWs are cleaned once a day or as needed which would seem inadequate based on our observation. Our current PME cleaning policy was based on CDC guidelines [14]. We observed PME (including COW) being cleaned in the patient room, or immediately outside the patient room, only 17 times in 144 h of observation. The sequence of touches, the network plot analysis, and the recovery of contamination on the COW implies a potential risk of transmission of pathogens directly from the COW to the patient via HCW hands. Although we observed less than desired levels of actions that reduce the transmission risk, our sequence data show that even if hand sanitization occurred 100% of the time at entry and exit, PME would likely still be contaminated from the touch sequences occurring during the care event. Considering the opportunities missed for hand hygiene in patient room and at exit along with contaminated PME moving from room to room, the transmission potential may further increase. COWs in our facility are required by policy to be disinfected at least once a day and as needed.

The network plots revealed that almost all the items touched were connected to at least a few other items in a sequence of touches. The patient, the most commonly touched item, had a potential for contamination from other surfaces as well as a potential for transmitting pathogens to other surfaces. Portable medical equipment have been found to be contaminated with MRSA, Vancomycin resistant enterococci and *Clostridium difficile* [19]. Thus, most items in the room have the potential to be contaminated if indeed the patient is considered the primary source of the contamination. If this is not in fact true, then the patient has the potential be become infected if the other objects are considered the source. Our results illustrating the interconnectedness of touch sequences between HCW and PME are consistent with published literature. While our study was not designed to evaluate hand contamination rates after touching the environment there have been previously published studies that have shown that HCW’s hands were contaminated almost as much by touching only the environment in the patient room as compared to when they touched both the patient and environment [22].

Our study had a few limitations: First, research staff did not record sequences that occurred outside of the patient room or in the bathroom. The majority of HCWs observed were from nursing staff. HCW’s may have modified their behavior because the observers were in the room; however, this possibility is mitigated by the fact that the HCWs agreed to being observed and seemed comfortable with their infection prevention activities. While these results do validate high-touch surfaces, our study was not designed to support implications for hand hygiene. We are pursuing further studies through participatory action research with our nurses to understand the implications of this interesting finding on daily practice.

## Conclusions

In conclusion, the surfaces that have been traditionally considered high-touch were identified as most frequently touched in our study. PME, such as a COW, emerged as a potential source for transmission of both ABC and MRSA. The disinfection of the COW between patients was not required by policy based on CDC guidelines; these guidelines may warrant reconsideration. Disinfection of PME, preferably in between patient interactions, may potentially be necessary, along with optimal hand hygiene, to reduce the possibility of transmission between patients. The current guidelines need to be reviewed using empirical data.

## Additional file

Additional file 1: Room Observation Template. (XLSX 11 kb)
